# Magnetic Resonance Imaging of Temporomandibular Joint and Aortic Root Score in Fibrillinopathies

**DOI:** 10.3390/medicina60101572

**Published:** 2024-09-25

**Authors:** Paloma Moisii, Alexandru Gratian Naum, Andra Mara Ursu, Adrian Vilcu, Irina Esanu, Irina Jari

**Affiliations:** 1 1st Medical Department, “Gr.T.Popa” University of Medicine and Pharmacy, 16 Universitatii Street, 700115 Iasi, Romania; irina.esanu@umfiasi.ro; 2 “Promedicanon” Cardiology Office, 15 Valea Prisacii, Valea Lupului, 707410 Iasi, Romania; 3 2nd Morpho-Functional Department, Biophysics and Medical Physics, “Gr.T.Popa” University of Medicine and Pharmacy, 16 Universitatii Street, 700115 Iasi, Romania; 4 “Neolife” Medical Center, 52 Carol I Avenue, 700503 Iasi, Romania; 5 Radiology and Medical Imaging Clinique, “Sf.Spiridon”Emergency Hospital, 1st Independentei Avenue, 700111 Iasi, Romania; dr.andraursu@gmail.com (A.M.U.); irina.jari@umfiasi.ro (I.J.); 6 Faculty of Industrial Design and Business Management, Department of Engineering and Management, “Gh.Asachi” Technical University of Iasi, 700050 Iasi, Romania; adrian.vilcu@academic.tuiasi.ro; 7 “C.F.” Hospital Iasi, Medical Clinic, 1 Garabet Ibraileanu Street, 700506 Iasi, Romania; 8 2nd Surgical Department, “Gr.T.Popa” University of Medicine and Pharmacy, 16 Universitatii Street, 700115 Iasi, Romania

**Keywords:** Marfan Syndrome, MASS syndrome, mitral valve prolapse syndrome, Marfan-like skeleton features, aortic Z score, temporomandibular joint dysfunction, disc displacement

## Abstract

*Background and Objectives:* Fibrillinopathies have different phenotypic expression: Marfan-like skeleton features, MLSF; mitral valve prolapse syndrome, MVPS; MASS phenotype, M = mitral valve prolapse, MVP, A = aortic root dilation, S = skeletal features, and the second S = (cutaneous) striae; Marfan Syndrome, MS. The study had the following main objectives: the correlation between disc displacement, DD (a major sign for temporomandibular joint dysfunction, TMDs) on magnetic resonance imaging, MRI, and aortic Z score (a major sign for aortic root dilation) on echocardiography; the predictive value of DD towards aortic Z score. DD had 2 types of severity: DD with reduction (the mild type, DDwR), and DD without reduction (the severe type, DDwoR). *Materials and Methods:* The type of fibrillinopathy was precised by clinical exam (systemic score), ophthalmic exam (for ectopia lentis), and echocardiography (aortic Z score, MVP). Medical treatment consisted of betablockers, BB (for patients with tachycardia), or angiotensin II receptor blockers, ARB. Surgical treatment was addressed to aortic root aneurysm, and severe mitral regurgitation, MR. DD benefited from dental conservatory treatment or surgical restoration. *Results:* DD-Z score had a powerful correlation in MASS (Rho = 0.787, *p* < 0.01), and in MS patients (Rho = 0.819, *p* < 0.01). For the entire sample, both DDwR-Z score and DDwoR-Z score had a moderate correlation (Rho = 0.469, and respectively 0.669). Furthermore, DD was an important predictor for Z score. DDwoR had a double power of prediction for the Z score compared with DDwR (B coefficient = 1.661 for DDwR and 3.281 for DDwoR). *Conclusions:* TMDs had a powerful correlation with aortic root dilation in MASS and MS patients from the sample. Likewise, TMDs was a major predictor for aortic root dilation, in the entire sample. In clinical practice, we can utilize an extracardiac finding, TMDs, obtained by a non-invasive technique, MRI, for cardiac severity stratification of fibrillinopathies.

## 1. Introduction

Fibrillinopathies are different phenotypic expressions of FBN1 encoding gene mutation. They range from mild conditions to the most severe, MS. MLSF expresses skeletal findings, without cardiovascular or ocular manifestations [1]. This is the mildest type of fibrillinopathy, and the patient’s prognosis is good. The consequences of MLSF are related especially to psychological distress due to skeletal modifications and joint pain.

MVPS is another fibrillinopathy, with skeletal and cardiovascular abnormalities. MVP is the central finding of this syndrome. Myxomatous degeneration of the mitral valve is a consequence of abnormal fibrillin, and this degeneration leads to the prolapse of mitral leaflets [2,3].

The third type of fibrillinopathy is the MASS phenotype. MASS involves the aortic root, with a borderline dilation [1]. The enlargement of the aortic root diameter is not as severe as in MS, and MVP usually has a mild/moderate expression in MASS.

MS is the most severe fibrillinopathy due its cardiovascular impact. MR can be severe in MS, with hemodynamic implications and can require surgical correction. Aortic root dilation in MS can be complicated by aortic aneurysm, aortic dissection, or rupture [4,5]. International guidelines highlight the importance of systemic features and echocardiographic findings, for the correct diagnosis of fibrillinopathies. Loeys et al. established the revised Ghent criteria in 2010. These guidelines highlight aortic root dilation and ectopia lentis as cardinal features in MS. The systemic score, according to these criteria, comprises skeletal, facial, lung, dura, mitral valve, and skin modifications [6].

There are not estimates regarding the incidence of MLSF, MASS, and MVPS. Only the incidence rate of MS is estimated: 1 in 5000 persons, according to several studies [7,8,9,10]. However, the real incidence of MS is underestimated. The phenotype is expressed after the first decade of life, and children below 10 years can be underdiagnosed [11,12]. Prevalent systemic features, like pectus carinatum or excavatum; wrist sign; or cutaneous striae significantly increase at 15–17 years. Aortic root diameter and ectopia lentis remain stable during the first two decades of life [13,14]. Four decades ago, mean life expectancy with MS was 32 years [15]. Improvement in cardiovascular treatment (medical, and surgical) has doubled the life expectancy of patients with MS [16].

Apart from the classical features previously mentioned, MS has other modifications that can worsen the outcome. Pulmonary artery dilation has been observed in half of MS patients, and the rupture of a pulmonary artery aneurysm can cause death [17]. Intrinsic cardiomyopathy [18,19] and severe ventricular arrythmias in MS can represent a significant cause of death [20].

Increased tortuosity of aortic branch arteries (vertebral, carotid, subclavian, iliac arteries) suggests an aggressive form of MS [21]. Tricuspide valve prolapse (TVP) is another marker for a severe disease; significant aortic root dilation, severe mitral valve prolapse are frequently noticed when the patient has TVP [22].

The first class of medication in MS is represented by BB, which improved aortic stiffness and elasticity [23]. Angiotensine converting enzyme inhibitors, ACEI, are an alternative treatment to BB, and this medication is proven to diminish aortic stiffness [24]. ARB can have effects on aortic root and clinical events in MS that are comparable with the effects produced by BB. Although ARB are not superior to BB in monotherapy in MS patients, they might be an alternative to BB, especially when BB are not tolerated or are contraindicated [25].

Surgical treatment is recommended when the aortic root diameter exceeds 50 mm in adults, or when the aortic Z score is above 4 in children. Usually, the David procedure and the Bentall procedure are preferred in aortic root surgery in MS [26]. Surgical repair of the aortic root is similar with the techniques utilized in other types of aortitis—Takayasu’s arteritis [27], syphilis, systemic erythematous lupus, rheumatoid arthritis, and sarcoidosis. Severe MR, with hemodynamic consequences (pulmonary hypertension) requires surgical repair. The majority of severe MR can be restored by cardiac surgery; only a small number of MS patients require mitral valve replacement [28].

TMDs is a common disorder, with impact on quality of life. In the global population, TMDs incidence is 34%; in Europe, is almost an equal distribution of males and females. This disorder is common between 18 to 60 years [29]. The prominent symptom in TMDs is pain in the jaw, temple, or ear, modified with jaw movements. The signs in TMDs are clicking during jaw movements, and pain after palpation of masseter/temporalis muscles [30].

TMDs is an extracardiac modification of MS, usually underestimated; its symptoms and signs are noticed in almost half of MS [31]. MRI increases the prevalence of TMDs from 50% to 81% in MS [32]. The MRI diagnosis of TMDs can include DDwR and DDwoR as major findings. De Stefano et al. noticed the association between generalized joint hypermobility and MRI diagnosis in TMDs [33,34]. Their studies prompted us to investigate the correlation between TMDs and major cardiac findings in fibrillinopathies, as generalized joint hypermobility is a common feature in these diseases.

The main objective of our study was to establish the relationship between TMDs, an extracardiac finding, and aortic root score in MS, MASS, and MVPS. Abnormal fibrillin is a protein located all over the connective tissue in fibrillinopathies. We investigated the hypothesis that temporomandibular joint modification in fibrillinopathies has similarities with aortic root alteration. TMDs was confirmed by MRI, and disc displacement, DD, was the major imagistic finding. Aortic root alteration was quantified by echocardiography, through aortic Z score. An anti-hypothesis can be the following: there is no correlation between TMDs and aortic root dilation. The predictive power of DD towards the aortic Z score was also included in the main objective of our research. An anti-hypothesis of it can be the following: DD has no predictive value towards aortic root dilation.

The secondary objectives of the research were the following: the correlations between echocardiographic parameters, the discreet association with relevance for the prognosis of these diseases, and the association between job satisfaction, and SS. The anti-hypothesis for secondary objectives are the following: echocardiographic parameters are not correlated in fibrillinopathies; the prognosis of these diseases has no discreet association with DD; job satisfaction has no correlation with SS.

## 2. Materials and Methods

### 2.1. Study Design

The study was conducted in accordance with the rules, and principles of evidence-based medicine, in compliance with the requirements of the Declaration of Helsinki of the World Medical Association 2013, and was approved by the Committee of Ethics of “Gr.T.Popa” University of Medicine and Pharmacy, protocol no 206, dated 30 June 2015.

The recruitment of patients, the investigations, and their treatment was completed between August 2015 and August 2016. The research type was a retrospective cross-sectional study. The study comprised 83 patients diagnosed with fibrillinopathy. The entire sample was divided into four groups, according to phenotype expression. The first group included 24 patients with MLSF, the second group comprised 22 patients with MVPS, the third group had 16 patients with MASS, and the fourth group had 21 patients with MS.

### 2.2. Inclusion Criteria

The diagnosis and the treatment protocol were explained to each patient, including pediatric patients. All patients and their parents (for pediatric patients) were informed about the benefits and risks of participating in this research. An informed consent approved by the ethics committee was signed by adult patients, or by their parents for pediatric patients. This consent also included permission for publishing the data in present and future research papers. The inclusion criteria were the revised Ghent criteria for the diagnosis of MS and related conditions, which are detailed in Section 2.3 Methods section [6].

### 2.3. Methods

#### 2.3.1. Systemic Score

The following signs were investigated, and the sum of the points given to each sign was the systemic score, SS [6]:
**Sign****Points**Wrist and thumb sign3Wrist or thumb sign1Pectus carinatum deformity2Pectus excavatum or chest asymmetry1Hindfoot deformity2Plain pes planus1Pneumothorax2Dural ectasia2Protrusio acetabuli2Reduced upper/lower skeleton and increased arm/height and no severe scoliosis1Scoliosis or thoracolumbar kyphosis1Reduced elbow extension1Facial features (3/5): dolichocephaly, enophtalmos, downslanting palpebral fissures, malar hyoplasia, retrognathia1Skin striae1Myopia > 3 diopters1Mitral valve prolapse1

#### 2.3.2. Transthoracic Echocardiography

Echocardiography was made with Fukuda Denshi 850XTD, using B-mode, color, and color Doppler. The aortic Z score was the parameter for severity of aortic root involvement. The aortic root diameter was measured at the level of sinuses of Valsalva, during the end of diastole. We calculated the Z score with the following equation: Z score = (aortic root diameter − estimated aortic root diameter)/0.24. Estimated aortic root diameter = 1.12 × body surface. Aortic root diameter was expressed in cm and body surface in m2 [35].

Mitral valve prolapse, MVP, was diagnosed according to the definition of the European Association of Echocardiography. We utilized a long axis parasternal view. MVP meaned more than 2 mm displacement of the leaflets into the left atrium. This had to occur during systole. The mitral leaflet thickness had to be more than 5 mm in MVP. Mitral regurgitation, MR, associated with MVP, was assessed by color Doppler. We utilized parasternal long and short axis views and apical long axis 2 and 4 chambers views, for MR assessment. Severe MR was defined by quantitative methods: color Doppler jet area, and vena contracta (the smallest region of the color jet at the regurgitant orifice) [36]). Severe MR had a color Doppler jet area > 60% of left atrium area, and a vena contracta width ≥ 7 mm [37,38].

#### 2.3.3. Fibrillinopathy Diagnosis

Ectopia lentis was the displacement of the crystalline lens, and was diagnosed by the ophthalmologist. Only MS patients had this medical condition. The Z score, and SS were diagnosed by the cardiologist. Table 1 summarized the diagnosis criteria utilized in our study, for different phenotypes.

#### 2.3.4. TMDs Diagnosis

Clinical TMDs diagnosis was established by a dentist, according to the symptoms, and signs discussed in the Introduction. The dentist used research diagnostic criteria for temporomandibular disorders, RDC/TMD questionnaires [33]. The patients with clinical TMDs were referred to MRI, and scans were made with a Philips 1.5 Tesla MRI machine(Manufacturer Philips, city Ravensburg, country Germany). Sagital and coronal projection were used, with sections made every 3 mm. The images were obtained in complete occlusion of opposing teeth, and in open mouth. The evaluation of the articular disc with MRI confirmed two types of disc displacement, DD, in TMDs: DDwR, and DDwoR. In DDwR, the articular disc was displaced in closed mouth position. In open mouth position, the disc reestablished the normal position relative to the condyle, in DDwR. The other type of DD, so-called DDwoR, had the following features: the patient had DD, in both open and closed-mouth positions, and the open mouth position could not restore the correct relationship disc-condyle (as in DDwR).

#### 2.3.5. Treatment 

MS, MASS, and MVPS patients were advised to avoid isometric exercises such as weight training, and high-resistance activities that activate the Valsalva maneuver.

Medical treatment was recommended for patients with MS, in order to control aortic root involvement. They received BB, Bisoprolol 2.5–5 mg twice daily (11 patients with tachycardia), or ARB, Telmisartan 40–80 mg daily (10 patients).

Open surgical reconstruction of the aortic root was recommended for aortic root dilation > 50 mm in adults, or in Z score > 4 in children. All of these MS patients with surgical indication for aortic root dilation also had symptomatic severe MR, and they undergone mitral valve surgery.

DDwR benefited from dental conservatory treatment, and DDwoR underwent surgical treatment for DD.

#### 2.3.6. Job Strain Score in Employees with Fibrillinopathies

We utilized a specific questionnaire: satisfaction with work scale (SWWS) [39]. The questions addressed the level of satisfaction in the workplace, and the answers were scored from 1 to 5. A Likert score of 1 meant that the patient’s response to the affirmations was “totally disagree”, so he/she had severe dissatisfaction with the workplace. A Likert score of 2 meant “partially disagree”, 3 was “almost agree”, 4 meant “agree”, and 5 meant “totally agree” with the affirmations.

#### 2.3.7. Statistical Analysis

Statistical analysis was made with IBM SPSS Statistics, version 22. To calculate the sample size (n = 83 patients) with a confidence level (*p* = 95%) and margin of error (e = 11%), we used Cochran’s theorem [40]. A Kolmogorov–Smirnov test revealed that statistical variables as age, SS, and Z score did not have a normal distribution; non-parametric statistical tests were suitable in our study. Correlations between Z score and DDwR/DDwoR/TVP/MR/AR were established with a Spearman test. Spearman’s correlation coefficient, Rho, was calculated with non-parametric bivariate correlation, from SPSS v22. Rho < 0.3 signified weak correlation; 0.3 ≤ Rho < 0.7 was a moderate correlation, and 0.7 ≤ Rho ≤ 1 was a powerful correlation. *p* value < 0.05 signified statistical significance, and *p* value < 0.05 meant high statistical significance. For the association between DD (both DDwR, and DDwoR) and Z score, for the entire sample, Smart PLS v 4.1.0.4 programming was necessary. Linear regression analysis was applied for investigate the predictive power of DDwR and DDwoR towards the Z score. Clustering analysis used the K-Means Clustering Algorithm; it clarified subtle associations, which is important for the disease prognosis.

## 3. Results

### 3.1. The Clinical Characteristics of the Entire Sample

The entire sample had similar numbers of females and males, which also applied to the MLSF, MASS, and MS groups specifically. Only the MVPS group had a predominance of females. Gender disparities were noticed in aortic aneurysm prevalence. Men had a higher prevalence of aortic events (aortic aneurysm, for our research) than women in our research (among 11 patients with surgical aortic root indication, 8 were men: 72% of surgical patients).

Mean age for the entire sample was 20.9 years, with 8.8 SD. The youngest patient was 8 years old, and the eldest was 45 years old. Age was implicated in the prevalence of aortic root dilation. Adults with MS had a higher prevalence of this finding (among 21 patients with aortic root dilation 61% were adults), compared with child patients with MS (among 21 patients with aortic root dilation, 39% were children). Age was not implicated in the prevalence of aortic events for MS patients: 6 adults and 5 children required surgical intervention (the number of adults with aortic aneurysm were equal to the number of children with this condition).

The most frequent findings were cranio-facial modifications (83.1%). Other common signs were the following: chest deformity (81.9%), MVP (71%), cutaneous striae (69.8%), foot deformity (67.3%), and wrist ± thumb sign (43.3%). TMDs were diagnosed in 24 patients (28.9%), and only these 24 patients underwent MRI for imaging diagnosis of TMDs. The mean aortic Z score was 1.88 ± 1.18 (minimum = 0.7, and maximum = 4.78). The mean SS was 6.36 ± 3.61 (minimum = 3, and maximum = 16). More than half of our patients had a family aggregation of fibrillinopathies. These observations are summarized in Table 2. 

### 3.2. The Relationship between TMDs, and Z Score

The severity of TMDs was established by MRI, meanwhile aortic Z score was quantified by echocardiography. Disc displacement was the major MRI finding for TMD.

None of the MLSF patients revealed DD on MRI, so this group was not included in DD and Z-score correlation.

Both in MVPS, and in MASS patients, TMDs was expressed only by DDwR (none of the patients from these groups had DDwoR). Statistical analysis revealed that in MVPS patients, the correlation DDwR-Z score was weak: Rho = 0.276, and without statistical significance: *p* value = 0.213. For MASS, the associated DDwR-Z score was powerful: Rho = 0.787, and with high statistical significance, *p* value < 0.01.

Among the MS group (21 patients), nine patients (42.8%) had DDwoR (severe DD), and six patients (28,5%) had DDwR (mild DD). The prevalence of TMDs (both types of DD) among MS patients was 71.3%. For MS patients with DDwoR, the related DDwoR-Z score was powerful: Rho = 0.819, and with high statistical significance: *p* value < 0.01. For MS patients, with DDwR, the correlated DDwR-Z-score was weak: Rho = 0.143, without statistical significance: *p*-value > 0.05.

These observations suggested to us that TMDs can be correlated with aortic root dilation in our study, but only in MASS and MS patients. In MASS patients, this correlation, the DD-Z score, was available for all of the patients; in MS patients, this association was available only for the patients with severe DD (DDwoR). These results are illustrated in Table 3.

**Figure 1:**
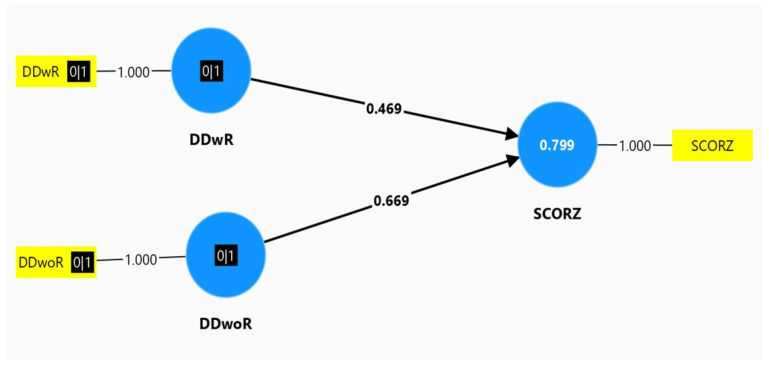
Smart PLSv 4.1.0.4. programming. Moderate correlation of 0.3 ≤ Rho ≤ 0.7.

We investigated the correlation between DD and Z score for the entire sample, and the results were the following: DDwR and Z score had a moderate correlation (Rho = 0.469, the superior arm, in Figure 1), compared with DDwoR and Z score, which had a moderate to powerful correlation (Rho = 0.669, the lower arm, in Figure 1). The number 0.799 above Z score signified the following: 79.9%, almost 80% of the sample, had a moderate correlation between DD and Z score. The statistical model was available for 80% of the entire sample, and this result is represented below.

### 3.3. The Predictive Value of TMDs for Z Score

We proved the following supposition: TMDs, expressed by DD on MRI examination, were an accurate predictor for aortic root dilation, expressed by Z score on echocardiography.

The linear regression analysis investigated the predictive value of DDwR and DDwoR, towards the dependent variable, Z score. The analysis was validated by the following parameters:

- R = 0.894, a powerful correlation (0.7 ≤ R ≤ 1) between DD and Z score
- R-square = 0.799, and Adjusted R-square = 0.794. All three of these parameters: R, R-square, and Adjusted R-square, confirmed that the statistical model explained the clinical supposition very well.

We confirmed that the regression model had statistical significance: *p*-value < 0.05. This is illustrated in Table 4.

Unstandardized coefficients, B, derived from this regression analysis allowed us to determine which DD type had the most influence towards Z score. Both B coefficients were positive and had statistical significance (*p*-value < 0.05). These results validated the fact that both DDwR and DDwoR had a predictive value for the Z score. Furthermore, B for DDwoR = 3.281, and this was an almost double value, compared with B for DDwR = 1.661. These results suggested to us that DDwoR had an almost double predictive value for the Z score, compared to the predictive power of DDwR for the Z score. These results are illustrated in Table 5.

### 3.4. Correlation between Echocardiographic Parameters

We investigated the association between the aortic root score and mitral regurgitation, MR. We noticed a powerful correlation (Rho = 0.817), with high statistical significance (*p* < 0.01), for the entire sample. For MASS and MVPS patients, this correlation, Z score-MR, had no statistical significance. For MS patients, the association had moderate significance (Rho = 0.442), with statistical significance for *p*-value (*p* = 0.045). These results are summarized in Table 6.

The association between aortic root score and aortic regurgitation, AR, revealed a moderate correlation: Rho = 0.536, with statistical significance *p* = 0.012, only for the MS patient group. The correlation between Z score and AR had no statistical significance for the entire sample, or in both MASS and MVPS patients specifically.

These results suggested us that both MR, and RA had a moderate and statistically significant correlation with Z score, only in MS patients. The severity of these valvulopathies is related to the severity of aortic root dilation in MS patients.

We correlated the Z score with another echocardiographic finding in MS patients: TVP. We noticed that the patients with TVP had higher values for aortic root dilation. A moderate correlation (Rho = 0.481) with statistical significance (*p* = 0.027) was noticed for the associated PVT-Z score. The PVT investigation in MS patients suggested to us the usefulness of this valvulopathy as a marker of severity for MS.

### 3.5. The Clustering Analysis

This specific method from statistical analysis permitted us to discover obscure associations that can be useful to identify disease evolution and severity. The variables utilized for this technique were the following: age, Z score, and SS. The adequate clustering model for these parameters comprised five clusters. Age was represented in blue column, Z score in green, and SS in beige. Mean value for variables was represented by 0, and standard deviation values were represented by 1, 2, and 3.

Patient distribution on the five clusters is represented in Table 7.

For the five clusters model we noticed the following:

- Cluster 1 had 17 patients with MLSF and 14 patients with MVPS. Low values for age, Z score, and SS were noticed in this cluster.
- Cluster 2 had 8 patients with MS. Low values for age, but the maximum values for Z score and for SS, were registered in this cluster.
- Cluster 3 had 13 patients with MS. High values for age (above 21 years), high values for Z score, and for SS (but not as high as in cluster 2) were noticed in this cluster.
- Cluster 4 had 15 patients with MASS. Low values for age (below 21 years) and middle levels for Z score and for SS were revealed in this cluster.
- Cluster 5 had 1 patient with MASS, 7 patients with MLSF, and 9 patients with MVPS. They were the “eldest” patients (the highest values for age); low values for Z score and SS were registered in this cluster.

**Figure 2:**
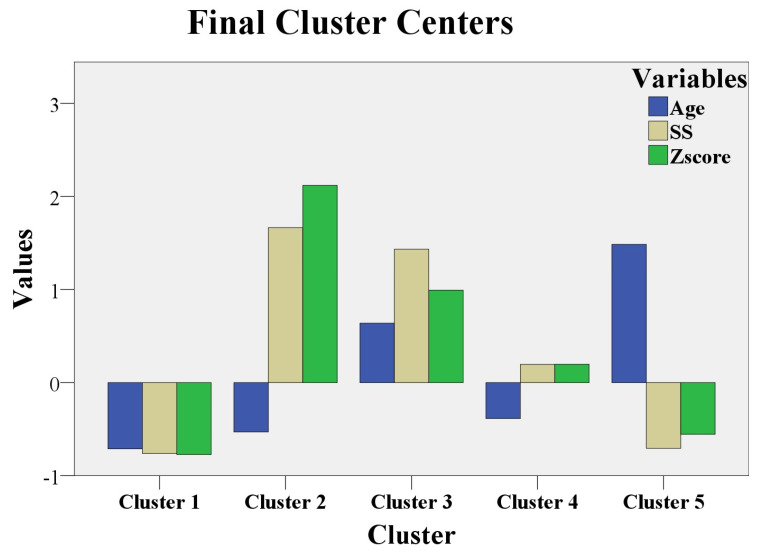
Clustering analysis.

All of these observations are illustrated in Figure 2.

This clustering analysis revealed the following observations:

- MLSF and MVPS (cluster 1 and cluster 5) had the lowest Z scores and SS, independent of age values. Figure 2 shows that Z score and SS in cluster 1 and cluster 5 were represented below 0 (mean = 0); the meaning of these results is that Z- score and SS had the lowest values for MLSF and MVPS patients. Age was below 0 (below mean age = 21 years) in cluster 1, with the youngest patients from the study. In cluster 5, age exceeded 1 (standard deviation = 1), and the patients were “the eldest” from the study (above mean age = 21 years). MLSF and MVPS patients were the youngest (cluster 1) and ‘’the eldest” (cluster 5) patients of our study.

These two groups, MLSF and MVPS had the lowest scores for both aortic and systemic involvement, independent of their age, which was the mildest expression in our study.

- The majority of MASS patient (15 of 16 MASS patients, 93%) had average levels of Z score and SS. MASS had a moderate expression in our study and the phenotypic findings were defined early, during childhood, or teenage; the majority of MASS, 93%, were aged below 21 years in cluster 4.
- MS patients with the maximum values for Z score, and maximum values for SS were children, or teenaged (cluster 2). These young MS patients (age is below 0, so patient age was below 21 years) had the most severe phenotypic expression of the fibrillinopathy. The other MS patients from cluster 3 had high values (but not as high as in cluster 2) for Z score and for SS; the 3thid cluster had “elderly” MS patients, aged above 21 years. This last category of MS patients (cluster 3) had a severe form of the disease, but not as severe as the first MS category (cluster 2). The youngest MS patients (cluster 2) had the worst prognosis in our study (the highest values for Z score, above 2, and the highest values for SS, above 1.5). The “eldest” MS patients had a severe prognosis, but not as severe as the youngest MS patients (high values for Z score and for SS in cluster 3, but not as high as in cluster 2).

### 3.6. SWWS Results

We investigated workplace satisfaction in 25 patients (the employees). They were teachers, engineers, and clerks. The MS patients (nine employees) had the worst Likert score: 1, and 2. These MS patients had severe dissatisfaction with their workplace. The other patients (16 employees) had a Likert score of 3; they had the following distribution: the group of MLSF had six patients, nine MVPS patients, and one MASS patient. These three groups had moderate satisfaction with their workplace. We noticed that the Likert score had a reverse correlation with SS. The correlation coefficient (r) value was −0.7 for all employee groups: r = −0.6 to −0.8 means negative strong association [30]. A high value for SS was associated with a severe dissatisfaction with the workplace.

## 4. Discussion

The study comprised 83 patients diagnosed with fibrillinopathy, with four different types of phenotypic expression: MLSF, MVPS, MASS, and MS. The main aim of the research was to investigate the association between an extracardiac finding—DD on MRI for TMDs—and a cardiac finding—Z score for the entire sample, and for different phenotypes. This is a peculiar aspect of the research, to investigate, and compare the results obtained for each phenotype. Usually, studies with fibrillinopathies investigate these phenotypes separately; MS or MVPS patients in particular are included in other research. Only a few studies include MASS or MLSF patients.

The incidence of these diseases was almost equal among men and women in the study, and these results are similar to those found in other research [41]. Although there were no gender differences for MS incidence, men had a higher prevalence for aortic dissection/aortic aneurysm in several studies [42,43]. We noticed the same gender disparities of aortic aneurysm prevalence in our research, just as in previously mentioned studies: a higher prevalence of aortic aneurysm in men compared with women. None of our MS patients evolved to aortic dissection.

The prevalence of aortic root replacement and mitral valve surgery in MS was similar in adults (six patients) and in children (five patients) in our research. Other studies noted disparities between childhood and adulthood surgical requirements: adults developed aortic events with a higher prevalence than children [44].

Aortic Z score was the central parameter in our study, and it was associated with all the other findings: DD on MRI; MR/AR/TVP on echocardiography. The mean value of the Z score was 1.88 for the entire sample. The highest values for Z score were in MS patients, the lowest values for aortic score were in MLSF patients, and medium values were noticed in MASS and MVPS patients. Other studies with MS patients noted values of their mean aortic Z score comparable with our mean Z score: mean Z score in Lopez et al. was 1.72 [45], in Pettersen et al. it was 1.23 [46], and in Gautier et al. it was 1.49 [47].

There was a high prevalence of pectus excavatum in MASS and MS patients. The correlation between aortic root dilation in previously mentioned disorders and pectus excavatum had no statistical significance in the study, as it was noticed by other researchers [48]. An interesting finding was noticed in pectus excavatum in otherwise healthy patients: a significant impairment of left ventricle function in transthoracic echocardiography; furthermore, left ventricular impairment was corrected after surgical repairment of chest deformity. The authors revealed that chest shape can induce the alteration of left ventricle kinetics, without an intrinsic myocardial dysfunction [49]. 

We selected the TMDs patients confirmed by MRI findings: anterior disc displacement, with and without reduction. The entire sample had the following distribution of TMDs: the MLSF group had no patients complaining of TMDs, the MVPS group had only one patient with TMD, the MASS group had 10 patients with TMDs, and the MS group had 15 patients with TMDs. The MLSF and MVPS groups diminished the prevalence of TMDs in the entire sample (31.2%). The prevalence of TMDs was very high in MS patients (71.4%) and high in MASS patients (38.6%). These results confirmed to us that TMDs was an important extracardiac aspect of our study. Other research investigated TMDs only in MS patients, and the prevalence of TMDs in MS was 81%, a close value to the percentage of TMDs prevalence obtained in our study in MS patients [50].

The main objective of our study was to establish the correlation between TMDs and Z score, and the predictive role of TMDs for Z score in fibrillinopathies. This is another peculiar aspect of the study. Other authors revealed a correlation between TMDs and quality of life, chronic pain, anxiety, and depression in MS [51], but no correlation between TMDs and Z score was investigated. Other researchers investigated the correlation between TMDs and generalized joint hypermobility, which was a common finding in fibrillinopathies, but no association between TMDs and Z score was discussed [33,34]. In our study, the correlation between TMDs and Z score was powerful in all MASS patients affected by TMDs (Rho = 0.787), and in MS patients (Rho = 0.819) with DDwoR; this correlation between TMDs and Z score was moderate for the entire sample (Rho = 0.469 for DDwR–Z score, and Rho = 0.669 for DDwoR–Z score). These results suggested to us that we can utilize an extracardiac finding, TMDs, for the assessment of cardiovascular severity in MASS and MS patients. DD on MRI was an important, and objective finding, provoked by abnormal fibrillin in the temporomandibular joint. Z score in echocardiography was another objective finding, addressed to aortic root dilation. This cardiac finding, aortic root involvement, was also provoked by abnormal fibrillin in the aortic layers, followed by reduced elasticity, and increased stiffness of the aortic root. The powerful association between DD and Z score has been noticed in MASS and MS patients, separately. When we investigated the DD–Z score correlation for the entire sample, we noticed a moderate correlation.

The predictive role of TMDs for Z score in fibrillinopathies was included in the main aim of the study. We proved with regression analysis that DD was a powerful predictor for Z score. Furthermore, DDwoR had an almost double predictive value for the Z score than the predictive power that DDwR had for the Z score. This result suggested us that the prognosis of cardiac involvement in fibrillinopathies could be stratified by an extracardiac finding: DD on MRI.

A secondary objective of the study was the association between echocardiographic parameters. The MR–Z score had a powerful correlation for the entire sample in our study. The severity of mitral valvulopathy was strongly correlated with the severity of aortic root dilation in the entire sample. In MS patients, both MR–Z score, and AR–Z score had a moderate correlation. The severity of both mitral and aortic valvulopathies was moderately correlated with the severity of aortic root dilation in MS patients. The TVP–Z score had a moderate association only in MS. We can conclude that the presence of TVP in MS patients suggested a worse prognosis, and this result was also noticed by other authors [51].

The clustering analysis revealed important data about disease severity in fibrillinopathies. MLSF and MVPS patients had a mild expression of the disease, independent of their age. MASS patients had a moderate phenotypic expression. The most severe expression of the disease was noticed among the youngest MS patients.

Workplace satisfaction had a reverse correlation with SS in our study. The patients had low scores for job satisfaction if their physical appearance (cranio-facial modifications, long upper arms, chest deformities, kyphosis, and other findings from SS) was different from their peers. Physical appearance was more important in our research, for the patients, than cardiac involvement severity. This result can be explained by the mean age of our patients: 21 years; for young patients, their physical appearance decreased their self-confidence and their perception about workplace. In other studies, the severity of cardiac involvement was an important determinant of job satisfaction [52,53]. The study had several limitations. One of the limitations was the absence of a longitudinal study, with different stages for investigation. Additionally, the genetic analysis was not considered in the study. A cardiac MRI was not performed in the study. This investigation could suggest an intrinsic cardiomyopathy. This cardiac finding was noticed in half of MS patients in other studies, and consisted of increased left and right ventricle end diastolic volumes and impaired systolic and diastolic function of both ventricles. Another limitation of the study was a discussion about life threatening ventricular arrythmias. These findings were noticed in MS in other researches, and could represent an important cause of death.

## 5. Conclusions

A peculiar aspect of this study was the inclusion of four different phenotypes of the same genetic disorder, FBN1 gene mutation: MLSF, MVPS, MASS syndrome, and MS. An extracardiac finding, TMDs, was strongly correlated with aortic dilation in MASS and MS patients. Furthermore, DDwoR on MRI, had a double predictive value towards aortic dilation, compared with the predictive value of DDwR. TVP was correlated with aortic root dilation. The most severe expressions (cardiac and extracardiac) were found in the youngest patients with MS. Workplace satisfaction had a reverse correlation with SS in MS patients. The severity of cardiac findings had no influence on job satisfaction.

Future directions of our study will include an investigation with a 5-year follow up of patients with fibrillinopathies. We will especially emphasize MASS and MS patients, as they have important cardiac involvement. Every 6 months, an assessment of aortic Z score, MR, AR, and SS will be performed and compared, in order to establish the evolution of cardiac findings. A comparison between echocardiography and cardiac MRI will be another future direction, for a precise assessment of intrinsic cardiomyopathy. Pain symptoms due to arthritis, chest deformities, bony overgrowth, scoliosis, kyphosis, and their implications for the occurrence of anxiety and depression, will be another future direction for our research.

## Figures and Tables

**Table 1:** Diagnosis criteria in different clinical aspects of the fibrillinopathy [6].

Disease Type	Z Score	SS	Ectopia Lentis
MS	≥3 (children)/≥2 (adults)	-	Yes
MS	≥3 (children)/≥2 (adults)	≥7	No
MASS	<3 (children)/<2 (adults)	≤5	No
MVPS	<3 (children)/<2 (adults)	<5	No

**Table 2:** Characteristics of the entire sample.

Characteristics	n	%	Mean ± SD
Sex - females	43	51.8	-
- males	40	48.2	-
Age	-	-	20.9 ± 8.8
Wrist ± thumb sign	36	43.3	-
Chest deformity	68	81.9	-
Foot deformity	56	67.4	-
Cutaneous striae	58	69.8	-
Cranio-facial aspects	69	83.1	-
Aortic Z-score	-	-	1.88 ± 1.18
Systemic score	-	-	6.36 ± 3.61
MVP	59	71	-
TMD	26	31.2	
Family medical history	45	54.1	-

n = number of patients; SD = standard deviation.

**Table 3:** Correlation between TMDs and Z score.

Disease Type	DDwR: n (%)	DDwoR (n, %)	Rho ^†^ (Zscore-DD)	*p* ^†^ (Zscore-DD)
MVPS	1 (4.5%)	-	0.276	0.213
MASS	10 (19.2%)	-	0.787 **	<0.01 **
MS	6 (7.2%)	-	0.143	0.536
MS	-	9 (10.8%)	0.819 **	<0.01 **

^†^ Spearman test; *p* < 0.01 ** high statistical significance; Spearman’s correlation coefficient, Rho; 0.7 ≤ Rho ≤ 1 powerful correlation.

**Table 4:** ANOVA values for the regression model: variable Z score and predictors DDwR and DDwoR.

ANOVA ^a^					
Model	Sum of Squares	Df	Mean Square	F	*p*-Value
1	Regression	92,494	2	46,247 159,041	<0.05 ^b^
	Residual	23,263	80	0.291	
	Total	115,757	82		

^a^. Variable: Z score. ^b^. Predictors: DDwoR, DDwR.

**Table 5:** Coefficients of the regression model analysis.

Coefficients ^a^						
Model		Unstandardized Coefficients		Standardized Coefficients	T	*p*-Value
		B	Std. Error	Beta		
1	(Constant)	1205	0.072		16,717	<0.05
	[DDwR]	1661	0.140	0.601	11,824	<0.05
	[DDwoR]	3281	0.216	0.772	15,177	<0.05

^a^. Dependent Variable: Z score.

**Table 6:** Correlation between Z score and MR.

Disease Type	Rho ^†^	*p*-Value ^†^
All the patients	0.817 **	*p* < 0.01 **
MASS	0.244	*p* = 0.362
MVPS	0.094	*p* = 0.676
MS	0.442 *	*p* = 0.045 *

^†^ Spearman test; *p* < 0.01 ** high statistical significance; *p* < 0.05 * statistical significance. Spearman’s correlation coefficient; 0.7 ≤ Rho ≤ 1 powerful correlation; 0.3 < Rho < 0.7 moderate correlation.

**Table 7:** Pacient distribution on the five clusters.

	C1 (n)	C2 (n)	C3 (n)	C4 (n)	C5 (n)	Total n
Disease type						
MASS	0	0	0	15	1	16
MLSF	17	0	0	0	7	24
MS	0	8	13	0	0	21
MVPS	14	0	0	0	8	22

C1 = cluster 1; C2 = cluster 2; C3 = cluster 3; C4 = cluster 4; C5 = cluster 5; n = number of patients.

## Data Availability

The original contributions presented in the study are included in the article, further inquiries can be directed to the corresponding authors.
